# Multicentric reticulohistiocytosis: A case report with response to adalimumab

**DOI:** 10.1002/ccr3.2926

**Published:** 2020-06-22

**Authors:** Shirley Kim, Emily M. Khatchaturian, Luis Dehesa

**Affiliations:** ^1^ Department of Internal Medicine UCSF‐Fresno Fresno CA USA; ^2^ Department of Dermatology UCSF‐Fresno Fresno CA USA

**Keywords:** arthritis mutilans, autoimmune, multicentric reticulohistiocytosis

## Abstract

Although MRH can mimic rheumatoid arthritis, its ability to rapidly progress to arthritis mutilans and association with malignancy in up to 25% of patients warrant prompt recognition and treatment along with age‐appropriate malignancy work‐up.

## Graphical abstract

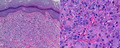

## 1 INTRODUCTION

Multicentric reticulohistiocytosis (MRH) is a rare systemic illness that presents with symmetric destructive arthritis in the setting of a cutaneous papulonodular eruption containing true histiocytes on biopsy. It is more likely to affect women (3:1), has a higher incidence in Caucasians, and is usually diagnosed between the patient's fourth and fifth decade of life.^1^, ^2^ Fifty percent of patients may also exhibit elevated inflammatory markers at diagnosis.^3^ There has yet to be an elucidated mechanism for the cause of MRH although some hypothesize a relationship to the activation of histiocytic receptor activator of nuclear factor kappa‐B ligand (RANKL) on the skin and joints.^1^

We present a case of multicentric reticulohistiocytosis (MRH), a rare systemic illness associated with destructive arthritis and papulonodular cutaneous lesions. Although our patient initially improved on prednisone and hydroxychloroquine, symptom recurrence following prednisone taper ultimately required biologics for control. Of note, an age‐appropriate malignancy screening work‐up was negative.

## 2 CASE SYNOPSIS

**FIGURE 1:**
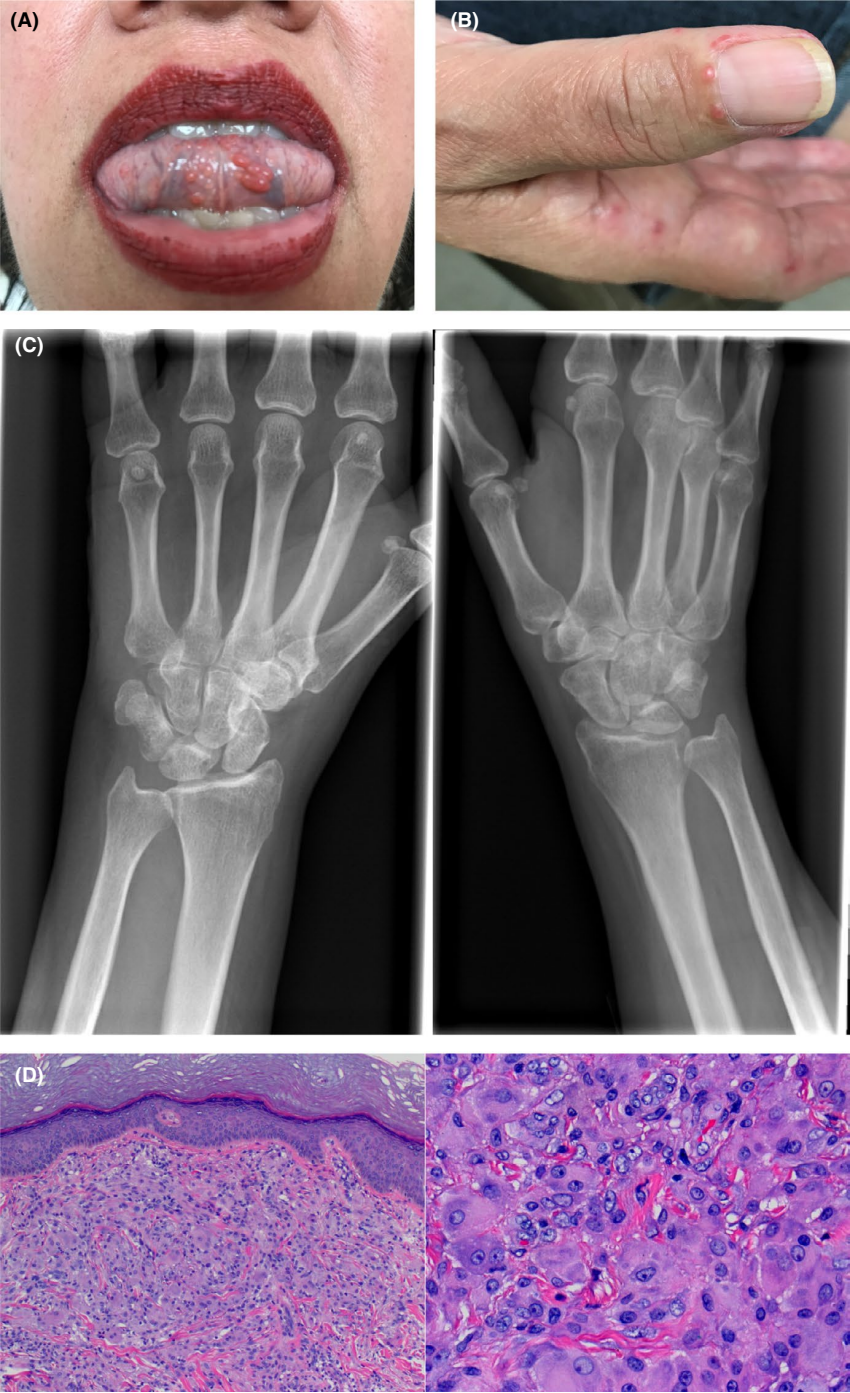
A. Multiple grouped, pink papules on the ventral aspect of the tongue. B. Multiple pink, firm, translucent periungual papules with a "coral bead" appearance. C. Bilateral wrist X‐ray revealing small, subchondral lucency underlying intact cortex. D. Punch biopsy of a papulonodular lesion showing diffuse dermal proliferation of epithelial histiocytes with abundant eosinophilic finely granular cytoplasm (a. hematoxylin‐eosin, original magnification 40×, b. 400×)

A 52‐year‐old woman with a history of hypothyroidism presented with 6 months of bilateral diffuse joint pain and malaise. She endorsed bilateral morning hand stiffness with swelling in her wrists, metacarpophalangeal (MCP), and distal interphalangeal (DIP) joints, knees, and shoulders. Additionally, she complained of difficulty raising her arms above her head. She developed an oral (Figure 1A) and periungual (Figure 1B) pruritic, papulonodular eruption followed by an erythematous patch along her neck that descended to her trunk. Topical triamcinolone and clobetasol relieved the pruritus but did not change the eruption's appearance. C‐reactive protein was elevated; however, infectious work‐up, ANA, rheumatoid factor, and anti‐CCP were all unremarkable (Table 1). X‐ray of her knee and wrist both showed small subchondral lucency underlying intact cortex (Figure 1C).

**Table 1:** ESR and CRP in parentheses decreased on three month follow‐up

ESR	11 (4)	RF	<10
CRP	21.6 (1)	ANCA	<1:20
ANA	Negative	Anti‐CCP	<16

## 3 MANAGEMENT AND OUTCOME

Biopsy of a papulonodular lesion revealed a nodular proliferation of mononuclear and multinucleated epithelial histiocytes with abundant eosinophilic finely granular “ground glass” cytoplasm (Figure 1D). The histiocytes were haphazardly dispersed between collagen bundles and extend into the deep dermis. The biopsy was consistent with multicentric reticulohistiocytosis. The patient's joint pain and cutaneous findings initially improved on a course of prednisone and hydroxychloroquine. However, she relapsed following prednisone taper. She was thus transitioned to adalimumab every other week with good control of cutaneous and joint findings. Of note, mammogram and CT of her abdomen and pelvis were unremarkable for signs of malignancy.

## 4 CASE DISCUSSION

Multicentric reticulohistiocytosis (MRH) is an exceedingly rare (fewer than 300 documented cases in the literature) systemic illness that presents with symmetric destructive arthritis in the setting of firm, round, reddish‐brown or flesh‐colored papules and nodules. The nodules can be scattered or grouped and are usually found along the acral regions of the body, face, hands, and juxta‐articular areas.^4^ MRH can also present with mucosal lesions in up to half of all cases.^2^, ^3^, ^4^ The papules are characteristically found periungually (coral beads) and in the nasolabial folds. Other features include leonine facies, periungual telangiectasias, pruritus, xanthelasmas, dystrophic nail changes, and a dermatomyositis‐like photodistributed rash.^2^ Although MRH may present with a dermatomyositis‐like rash, biopsy would reveal pathologic features of MRH as discussed below. Moreover, the hyperkeratotic, violaceous Gottron's papules found on the extensor surfaces of the DIP and MCP joints are distinctively different than the papulonodular rash of MRH.

Up to 25% of cases have an associated malignancy (breast, lung, gastrointestinal, hematologic, metastasis of unknown primary, etc); thus, it is of tantamount importance that patients diagnosed with MRH have a malignancy screening suitable for their age and past medical history.^3^, ^5^ Other organ systems that may be affected include the heart (pericardial effusions and myositis), lung (hilar adenopathy, infiltrates, fibrosis, and an association with Mycobacterium infections), lymph nodes, thyroid gland, gastrointestinal, and genitourinary systems.^3^ Systemic signs such as fevers, weight loss, fatigue, myalgias, and weakness can be seen.^1^

Rheumatoid arthritis (RA) and MRH have similar clinical pictures which may cause barriers to accurate diagnosis and rapid treatment. MRH usually affects DIP joints unlike RA and has a more insidious pattern of joint destruction.^1^ Radiographic images would show joint space narrowing in RA while in MRH there would be joint space widening.^5^ Another aspect that may confound accurate diagnosis is that 15% of cases of MRH have a coexisting autoimmune or connective tissue disease such as SLE, Sjogren's, or scleroderma.^2^, ^3^, ^6^ However, when MRH presents alone the autoimmune markers are typically negative.

Diagnosis of MRH is confirmed by histopathology of skin or synovium with hallmarks of multinucleated non‐Langerhans giant cells with ground glass eosinophilic cytoplasm.^5^ Biopsies are also PAS positive, CD 68 positive, TRAP positive, cathepsin K positive, S‐100 negative, and CD1a negative.^2^, ^3^

Of note, in a case report by Asano et al, when Doppler ultrasound revealed large bone erosions, the authors obtained an F‐fluorodeoxyglucose positron emission tomography/computed tomography (FDG‐PET/CT) with results suggesting an inflammatory process; biopsy ultimately helped establish the diagnosis of MRH. Although age‐appropriate malignancy screening with relevant imaging should be part of any MRH work‐up, this report highlighted the utility in joint ultrasonography as a helpful noninvasive imaging modality in diagnosing MRH.^7^

Treatment is critical due to rapid progression to arthritis mutilans. However, there are no established treatment guidelines due to insufficient patient population and lack of clinical trials. The most common treatment regimens usually include corticosteroids with methotrexate although MRH tends to be resistant to DMARDs. There has also been a recent interest in utilizing bisphosphonates and biologic agents such as TNF alpha inhibitors. There is spontaneous remission of most cases within 10 years of diagnosis.^1^

## 5 CONCLUSION

Multicentric reticulohistiocytosis is an exceedingly rare systemic illness that can mimic rheumatoid arthritis. Although there are no established guidelines for treatment, prompt recognition and treatment are crucial due to rapid progression to arthritis mutilans. Moreover, due to its association with malignancy, work‐up should include age‐appropriate malignancy screening.^8^

## CONFLICTS OF INTEREST

The authors declare no conflicts of interests.

## AUTHOR CONTRIBUTIONS

LD: conceived the idea. SK and EK: wrote the manuscript with revisions. The final manuscript was evaluated and approved by all authors.
